# Intrathoracic ureteric stent migration through a reno-pleural fistula: a case report of rare antegrade ureteric stenting complication

**DOI:** 10.1186/s12905-021-01405-2

**Published:** 2021-07-10

**Authors:** Yi-Hsuan Chen, Marcelo Chen, Yu-Hsin Chen

**Affiliations:** 1 Department of Urology, Mackay Memorial Hospital, Zhongshan Dist, No. 92, Sec. 2, Zhongshan N. Rd, Taipei, 104 Taiwan; 2 Department of Medicine, Mackay Medical College, New Taipei City, Taiwan; 3 Institute of Pharmacology, School of Medicine, National Yang Ming Chiao Tung University, Taipei, Taiwan

**Keywords:** Complication, Percutaneous nephrostomy, Fistula, Stent migration, Urinothorax

## Abstract

**Background:**

Malignant obstruction and associated hydronephrosis is a common complication of advanced cervical cancer. Percutaneous nephrostomy (PCN) followed by antegrade stenting is often required to relieve obstruction as retrograde access fails in considerable proportion of such patients. Reno-pleural fistula is a rare complication of PCN which creates a patent connection between the renal collecting system and the thoracic cavity, and urine accumulation in the pleural space can cause pleural effusion (i.e., urinothorax). Upward or downward migration is a complication of indwelling ureteric stents. Further migration with extrusion outside of the urinary tract is uncommon. Herein we present an unprecedented case in adult of ureteric stent upward migration through a reno-pleural fistula into the thoracic cavity managed by thoracoscopy.

**Case presentation:**

A 66-year-old female was diagnosed of advanced stage cervical cancer with suspicious bladder invasion. Given her bilateral hydronephrosis with impaired renal function, she underwent bilateral PCN and subsequent antegrade ureteric stenting. However, she presented with dyspnea, right back pain, and oliguria four days after bilateral PCN catheter removal. Computed tomography reported massive right pleural effusion and an intrathoracic ureteric stent within reno-pleural fistula. Thoracoscopy with thoracostomy was performed to remove the ureteric stent and urine in right pleural space. A week later, urinothorax had resolved and right PCN was performed again. She was discharged after regaining normal renal function with right PCN and a left ureteric stent in place.

**Conclusions:**

A reno-pleural fistula can serve as a route for ureteric stent migration and that continuous drainage of urine can cause urinothorax once the stent reaches the thoracic cavity. Anytime a supracostal approach is used for PCN, even when using small caliber catheters, clinicians should pay special attention given the risk of pleural injury and subsequent complications.

## Background

Women with cervical cancer or other gynecological malignancies often develop hydronephrosis, as a result of urinary tract obstruction form external compression of tumor, lymph nodes, inflammation, and scarring within the pelvis. This complication can be managed by ureteric stenting or urinary diversion, commonly percutaneous nephrostomy (PCN) [1].

Aside from the relief of urinary tract obstruction, indwelling ureteric stents are utilized in a variety of clinical settings including an adjunct to urolithiasis treatment, perioperative placement for ureteroscopic procedures, and as splints after ureteric anastomosis or injury [2]. Although the design of ureteric stents facilitates their ability to maintain an adequate position and function, stent migration is observed in 8–9.5% of patients [3].

PCN has a risk of pleural injury and subsequent hydrothorax or pneumothorax. Supracostal access is the primary risk factor that contributes to this complication, with an incidence rate as high as 10–30% above the 12th rib and 25–35% above the 11th rib [4]. Communication of the urinary tract and thoracic cavity can persist after the percutaneous tube is removed and can become a reno-pleural fistula. The continuous leakage of urine through the fistula leads to urinothorax, the accumulation of urine in the pleural space [5].

We present an extremely rare case of significant urinothorax resulted from intrathoracic migration of the ureteric stent through a reno-pleural fistula, and report its management by thoracoscopic intervention. To the best of our knowledge, this is the first-ever reported case of concurrent complications encompassing reno-pleural fistula and ureteric stent migration in adults. The diagnostic considerations and managements of such complications are also discussed.

## Case presentation

**Fig. 1:**
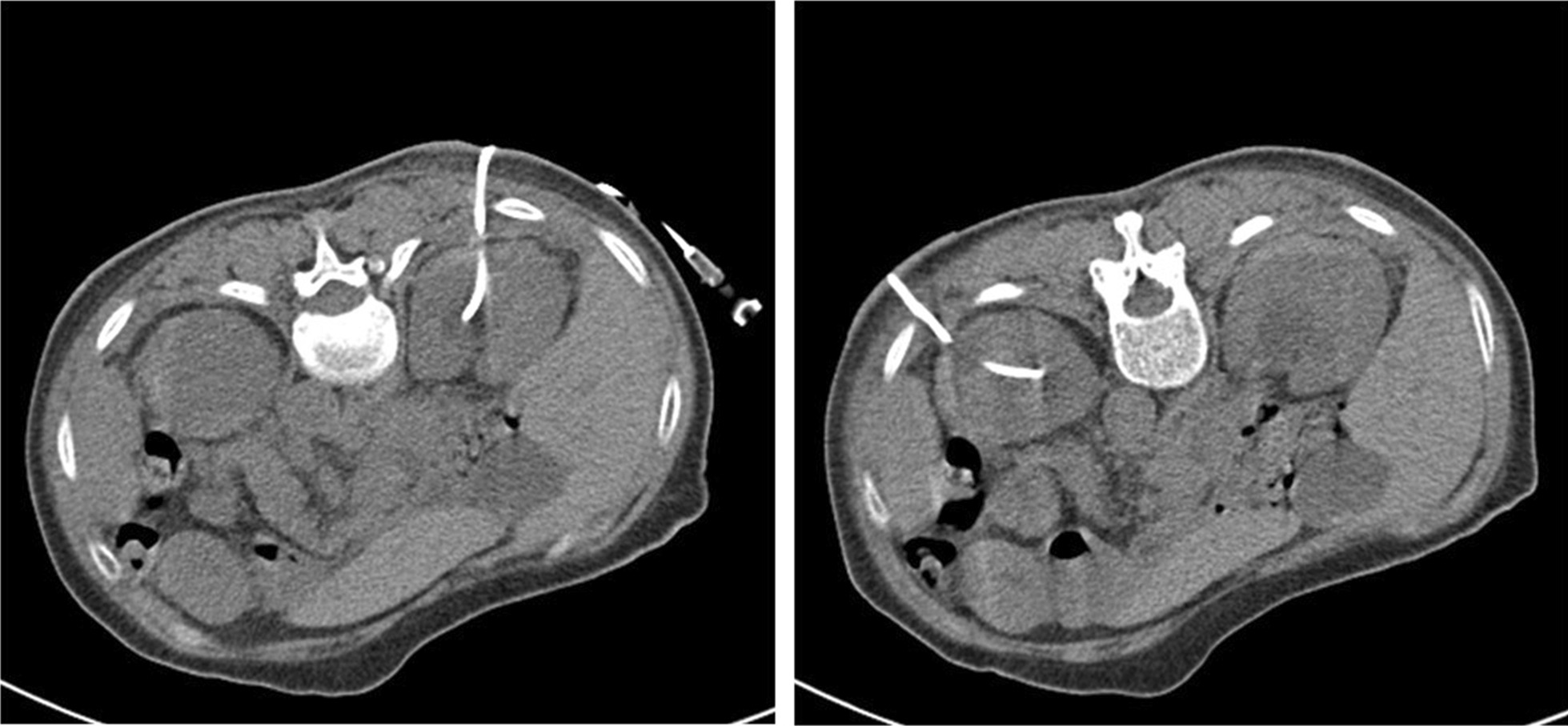
Bilateral computed tomography guided percutaneous nephrostomy with supracostal approach

**Fig. 2:**
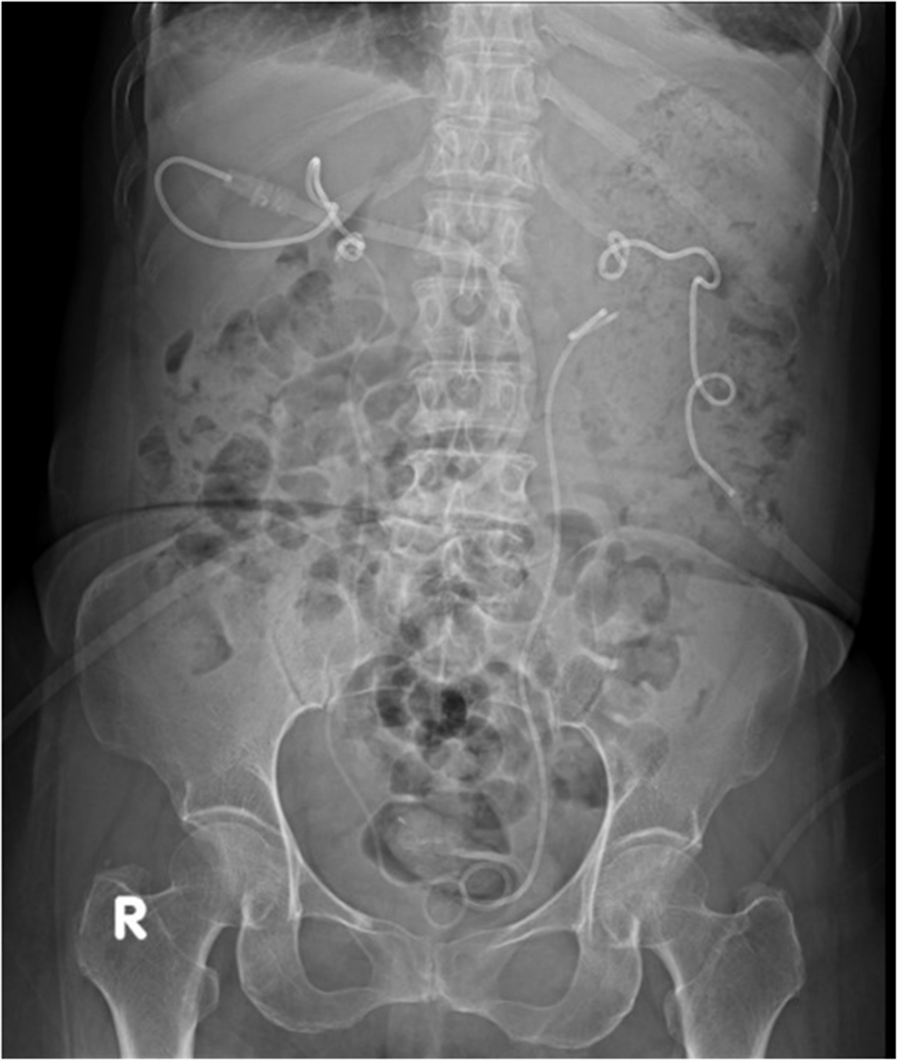
Bilateral percutaneous nephrostomy catheters and antegradely inserted ureteric stents. Note that catheter coils are entangled in right renal pelvis

A 66-year-old woman was generally well without known systemic disease or operation history. She visited emergency department due to post-menopausal vaginal bleeding for days. Transvaginal ultrasound revealed a pelvic mass about 7 cm in diameter, and computed tomography (CT) reported late-stage cervical cancer and bilateral hydroureteronephrosis from possible bladder invasion. The patient also complained of decreased urine output and her serum creatinine increased from 1.2 to 2.2 mg/dL. After admission, cystoscopy revealed tumor invasion into the bladder trigone and both ureteric orifices could not be identified. Biopsy of bladder trigone later proven to be invasive squamous cell carcinoma favoring cervical origin, which indicated a Stage IVA cervical cancer. As retrograde insertion of ureteric stent was not feasible, bilateral PCN with 6 Fr. Cope catheters and subsequent antegrade placement of 4.7 Fr. Double-J ureteric stents was performed by the radiologist under CT-guidance (Fig. 1). Bilateral supracostal approach of PCN was implemented to access more dilated renal calyces in higher-positioned kidneys, and antegrade ureteric stenting was performed 2 days later after hemostasis and verifying the patency of PCN by nephrostogram. In follow-up images the right PCN catheter was found to be entangled with the ipsilateral ureteric stent, and thus the patient underwent cystoscopy again during removal of the bilateral PCN catheters to confirm that the bilateral ureteric stents were in place (Fig. 2).

**Fig. 3:**
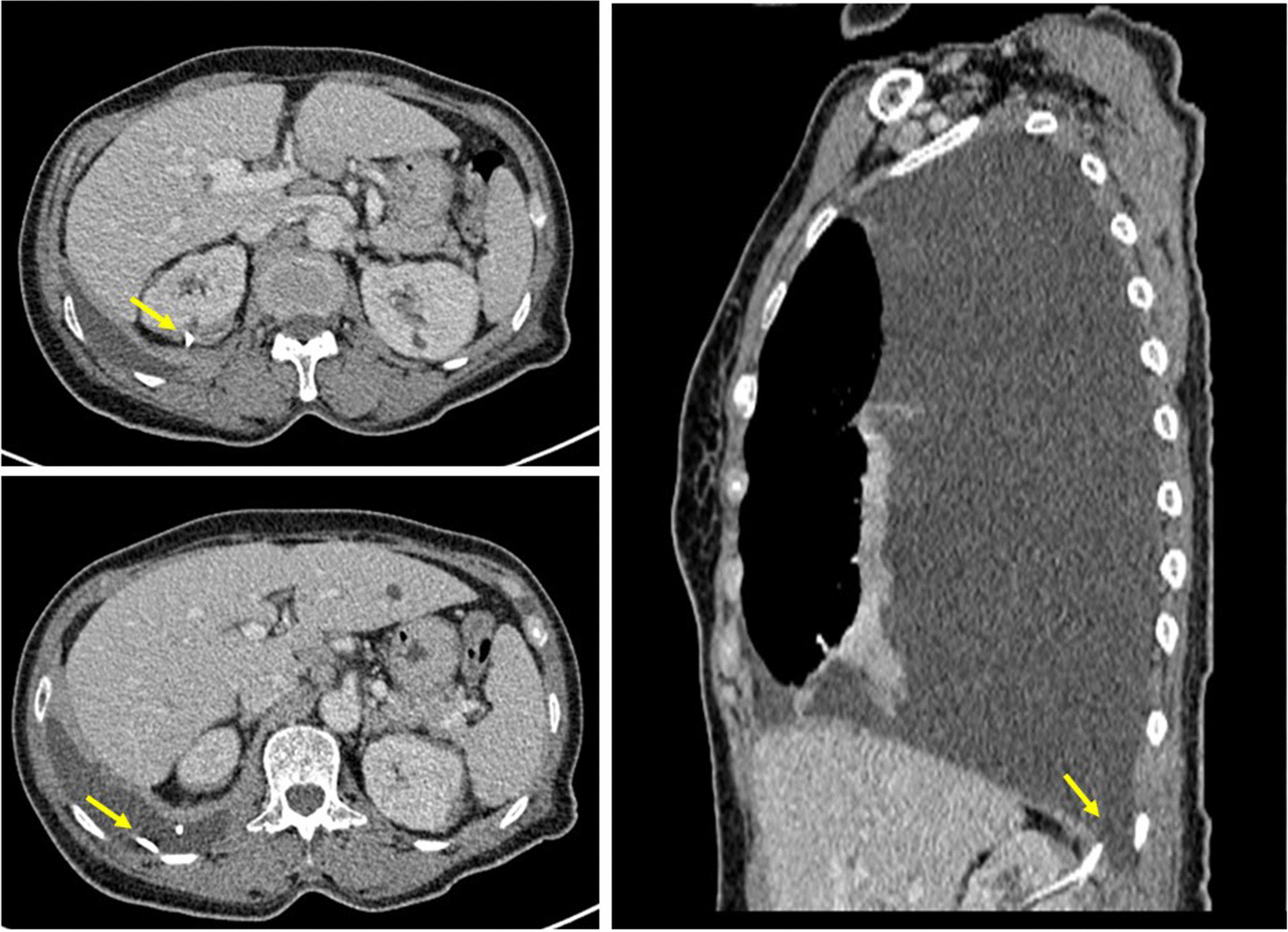
Migrated ureteric stent through reno-pleural fistula entering pleural cavity (arrow) and caused massive urinothorax

Four days after PCN catheter removal, the patient complained of severe right back pain accompanied by oliguria and dyspnea. A physical examination revealed right costovertebral angle tenderness. A chest CT showed the tip of the ureteric stent within the right basal pleural cavity, indicating intrathoracic migration of right ureteric stent which resulted in massive right urinothorax (Fig. 3). Reposition of the right ureteric stent was initially planned with cystoscope. However, only the left ureteric stent remained within the bladder and neither the right ureteric stent nor the ureteric orifice could be identified. This finding was compatible with upward migration of the right ureteric stent and it was not possible to perform a ureteroscopy. A thoracic surgeon was consulted for thoracoscopic management, and the intrathoracic ureteric stent was removed from the pleural space along with more than 2600 mL of bloody effusion. A chest tube was placed for drainage. The chest tube was removed a week later after resolution of the patient’s pleural effusion. Right PCN was performed again for persistent right hydronephrosis. The patient was then discharged after regaining normal renal function with right PCN and a left ureteric stent in place.

## Discussion and conclusions

Hydronephrosis or nonfunctioning kidney (unless known to be due to another cause) is one of the diagnostic criteria of Stage IIIB cervical cancer in the FIGO staging system [6]. It is associated with morbidities such as pain, nausea and vomiting, urinary tract infections, and renal failure. Patients of cervical cancer are also found to have worse survival if hydronephrosis presents [1], Previous literatures have reported that the placement of ureteric stent can restore or maintain renal function, but its impact on improvement of prognosis remains inconsistent across studies [6].

Most of hydronephrotic cervical cancer patients will undergo ureteric stenting if feasible, while widespread application of ureteric stents comes with inevitable complications. The innate characteristics of foreign body and vesicoureteral reflux can cause a constellation of symptoms, which are known as “stent syndromes”. These include irritative bladder symptoms, suprapubic discomfort, loin pain, and occasional gross hematuria [7]. Other more problematic complications, such as urinary tract infection, malposition, migration, obstruction, encrustation, or fractured stent, may occasionally occur and may require further intervention [8].

The occurrence of stent migration is multifactorial, and can be affected by ureteral peristalsis, the shape and material of the stent, and technical issues associated with its placement. A softer, more hydrophilic ureteric stent is, by its nature, more prone to migration. Distal migration is usually a result of failure of coiling in the kidney, which makes it unable to overcome ureteral peristalsis. Proximal migration is associated with stents which are too short, or erroneous and excessive pushing during their insertion [3]. Although it rarely happens, stent migration is not limited to within the collecting system. Extrusion of ureteric stents has been reported with a myriad of other destinations, including the abdominal cavity, the retroperitoneum, the pelvis, the vena cava, cardiac chambers, and even the pulmonary artery [3, 9]. Intrathoracic ureteric stent migration has been previously reported once in an infant after a bladder exstrophy repair [10], but to the best of our knowledge this complication has not been reported in adults prior to the current case.

Antegrade ureteric stenting from PCN is a well-established, safe alternative when retrograde placement is not feasible, especially in obstructive malignancies and when there is altered anatomy [11]. However, when using this approach there are additional risks which clinicians should be aware of. Hemorrhage, infection, visceral damage, urinary leakage, and pleural injury are notable complications which have been previously reported [12]. In rare cases, a fistulous communication can develop following pleural injury. It usually occurs after the removal of large-bore PCN for percutaneous nephrolithotomy, particularly if ureteric obstruction persists [13]. Batura et al. also demonstrated the formation of pleuro-renal fistula from PCN for antegrade ureteric stent placement [14].

To diagnose urinothorax from a reno-pleural fistula, a high degree of clinical suspicion is important. Acute onset of dyspnea, chest or flank pain, and decreased urine output in patients whose PCN catheter has been recently removed should be vigilantly assessed. Deteriorating renal function and rapid accumulation of pleural effusion in these patients implies the possibility of urinothorax. Retrograde pyelogram, contrast-enhanced CT, and renal scintigraphy are useful tools for identifying the presence of reno-pleural fistula [15, 16].

Thoracocentesis with biochemical analysis is often required for the diagnosis of urinothorax. A pleural fluid/serum creatinine ratio > 1 is regarded as the hallmark criterion but it has low specificity. Other characteristics include urine-like color or odor, transudative effusion by Light’s criteria, and a total pleural fluid protein < 1 mg/dL [15]. As the pleural effusion of our patient was apparently urine which flowed directly through the ureteric stent, the creatinine concentration was not examined. Bloody effusion was compatible with her hematuria, and it was also transudative with a low protein level.

Prompt recognition with multidisciplinary intervention is essential for the management of urinothorax. Decompressive measures for the underlying uropathy and drainage of the urinothorax with multiple thoracocenteses or thoracostomy are necessary to relieve pleural effusion and promote closure of the fistula [14]. In the present case, the cause of the urinothorax was the upward-migrated ureteric stent in the reno-pleural fistula. It was impossible to retrogradely retrieve the stent, and its intrathoracic location mandated thoracoscopic manipulation. Percutaneous access was the only remaining option for removal of the obstruction because it was not possible to identify the ureteric orifice.

The current case illustrates that a reno-pleural fistula can serve as a route for ureteric stent migration and that continuous drainage of urine can cause urinothorax once the stent reaches the thoracic cavity. To the best of our knowledge, this scenario has never been previously reported in adults. As retrograde ureteric stenting often fails in patients with cervical cancer or other obstructive malignancies, PCN with further antegrade ureteric stenting is widely performed in such clinical settings. Anytime a supracostal approach is used for PCN, even when using small caliber catheters, clinicians should pay special attention given the risk of pleural injury and subsequent complications. High index of suspicion for urinothorax should be maintained in patients developing dyspnea after PCN catheter removal, and rapid multidisciplinary managements could obtain excellent prognosis.

## Data Availability

The datasets used and analyzed during the current study are available from the corresponding author on reasonable request.

## Abbreviations

PCN: Percutaneous nephrostomy
CT: Computed tomography
mg/dL: Milligram/deciliter
mL: Milliliter
FIGO: International federation of gynecology and obstetrics
